# The Relationship Between Depression, Spiritual Well-Being and Spiritual Care Needs of Postpartum Women in Türkiye

**DOI:** 10.1007/s10943-025-02359-7

**Published:** 2025-06-12

**Authors:** Yasemin Erkal Aksoy, Hülya Türkmen, Bihter Akın

**Affiliations:** 1 Department of Midwifery, Health Sciences Faculty of Selçuk University, Konya, Türkiye; 2 Department of Midwifery, Health Sciences Faculty of Balıkesir University, Balıkesir, Türkiye

**Keywords:** Depression, Postpartum, Spiritual care, Women

## Abstract

This study was conducted to determine the relationship between depression, and spiritual well-being and spiritual care needs of women in the postpartum period. The Personal Information Form, Postpartum Depression Screening Scale, Spiritual Well-Being Scale and Spiritual Care Needs Inventory were used as data collection tools. A total of 430 postpartum women living in Türkiye were included in the study. According to the cut-off value of the Postpartum Depression Screening Scale, 36.5% of the participants were at risk for minor or major depression. There was a statistically significant negative relationship between the scores the participants obtained from the total Postpartum Depression Screening Scale and transcendence (devotion to a divine power) (r =  − 0.191, *p* < 0.01), harmony with nature (r =  − 0.200, *p* < 0.01), and anomie (meaning of life) (r =  − 0.463, *p* < 0.01). The Structural equation modelling analysis showed an acceptable model fit and revealed that higher levels of postpartum depression were significantly associated with lower levels of transcendence, harmony with nature and anomie. Due to the overlap between certain subscales of the Spiritual Well-Being Scale (harmony with nature and anomie) and mental health constructs, the correlations involving these subscales were not considered methodologically meaningful. As a result, only the relationships between the transcendent subscale, which specifically measures spiritual aspects, and postpartum depression were deemed significant.

## Introduction

The postpartum period is a transition period in which a newborn baby joins the woman's life, and she experiences physical and mental changes. Women may sometimes have difficulty adapting to this process (Asadi et al., 2022; Johansson et al., 2020). Secretion of hormones during and after birth is part of the psychological process that instinctively prepares the woman for motherhood. During the postpartum period, women are expected to adapt to their maternal role, to begin and continue successful breastfeeding, and to fulfill new tasks such as taking care of the baby.

However, some women have difficulty adapting to this period and thus they experience psychological problems such as stress and depression (Brummelte & Galea, 2016). Postpartum depression (PPD) is a disorder that has negative effects on the mother and baby. The prevalence of PPD is around 14%, and the prevalence is higher in developing countries (about 27%) (Hahn-Holbrook et al., 2018; Liu et al., 2022). In studies conducted in Türkiye, the prevalence of PPD has been reported to be approximately 24% among women in the postpartum period (Demir et al., 2016; Karaçam et al., 2018; Özcan et al., 2017).

Due to PPD, mothers are likely to suffer symptoms such as depressed mood, insomnia, loss of appetite, dizziness and irritability. Among PPD risk factors are genetic predisposition, low education level, gestational diabetes, history of depression, and lack of social support (Liu et al., 2022; Tolossa et al., 2020). PPD negatively affects mothers' ability to perform normal functions, mother-infant attachment, success of breastfeeding, quality of life, and marital adjustment (Çankaya & Ataş, 2022; Shimao et al., 2021; Stickel et al., 2021).

## Spirituality and Mental Health

The relationship between PPD and spiritual well-being may be bidirectional. Higher levels of spiritual well-being may contribute to lower levels of depression by fostering psychological resilience and providing emotional support (Akbari et al., 2020; Mosqueiro et al., 2021). Conversely, individuals experiencing lower levels of depression may be more likely to engage in spiritual practices, such as transcendence, harmony with nature, or religious rituals, as part of their coping mechanisms (Akbari et al., 2020; Gökçay & Aydın, 2024; Koenig & Carey, 2025). The concept of spirituality encompasses not only religiosity, participation in religious communities, and engagement in religious and spiritual practices but also personal activities such as relaxation techniques, meditation, and prayer (Koenig & Carey, 2024, 2025).

Spirituality is a multidimensional concept and is used in various contexts such as spirituality, spiritual goodness and spiritual well-being (Ekşi & Kardaş, 2017). In recent years, the definition of spirituality has broadened, encompassing diverse perspectives. It is essential to acknowledge individuals' self-defined spiritual experiences, which may extend beyond religious beliefs and practices (Garssen et al., 2021; Koenig & Carey, 2024, 2025).

Spiritual well-being is increasingly recognized as an integral component of mental health, encompassing psychological resilience, emotional balance, and a sense of meaning and purpose (Garssen et al., 2021; Zimmer et al., 2016). In several studies, it is indicated that there is an inverse relationship between spiritual well-being and PPD (Clements et al., 2016; Oxhandler et al., 2021). In addition, PPD symptoms are less common in women who are religious, whose spirituality is high, and who participate in religious organizations (Cheadle & Dunkel Schetter, 2018; Clements et al., 2016).

During the postpartum period, healthcare primarily focuses on women's physiological needs, as they are more easily assessed. However, individuals also engage in various spiritual practices and behaviors, such as transcendence, religious rituals, prayer, and a harmony with nature, which contribute to their spiritual well-being (Kirchoff et al., 2021; Seddigh et al., 2016). Therefore, alongside physical needs, the evaluation of spiritual dimensions should also be integrated into postpartum care to ensure a more holistic approach to maternal health.

Spiritual needs, on the other hand, have a more abstract and complex structure than physical needs. Health professionals should be aware of spiritual needs when they provide care (Ghorbani et al., 2021; Puchalski, 2021; Sun et al., 2021). By identifying women's spiritual care needs, health professionals can give prominence to individual care and thus reduce the risk of PPD. Although several studies have been conducted on the relationship between PPD and spirituality in different societies, Turkish literature lacks such studies.

Therefore, the relationship between PPD and spiritual well-being should be investigated in Turkish culture where most people have Islamic beliefs. The study aimed at determine the relationship between depression, and spiritual well-being and spiritual care needs of women in the postpartum period.

## Materials and Methods

### Study Design

The study has the descriptive correlational design.

## Research Variables and Questions

While the dependent variable of the study is the depression levels of women in the postpartum period, its independent variables are their descriptive characteristics, spiritual well-being levels and spiritual care need levels.

Research questions are as follows:

1. What are the levels of depression, spiritual well-being and spiritual care needs of women in the postpartum period?
2. Is there a relationship between depression and spiritual well-being and spiritual care need levels of women in the postpartum period?

## Participants

The study population consists of all women who presented to the gynecology outpatient clinic for postpartum check-ups and agreed to participate in the study conducted at a Faculty of Medicine in Konya/Türkiye between May 2022 and December 2023. The sample size of the study was calculated as 427 women with the G*Power 3.1.7 program based on the known score of the Spiritual Well-Being Scale (120.57 ± 11.43) within 2 points deviation (effect size: 0.17, power: 95%) (Özcan et al., 2020).

*Inclusion criteria*: Being ≥ 18 years old, having given birth within the last year, volunteering to participate in the study, not having a diagnosis of a mental disorder, being at least a primary school graduate, and being a Turkish citizen. *Exclusion criteria*: Not filling out the data collection forms completely and having a baby with a health problem. Considering the possibility of withdrawals and/or losses during the study, we decided to include 5% more women (N = 445). Of them, 15 who did not fill out the data collection forms completely were excluded from the study. Thus, the study was completed with 430 women.

## Data Collection

Women were recruited into the study using the convenience sampling method. Data were collected by the researchers based on self-report. The women were informed about the study at the outpatient clinic and requested to participate in the study. Of the women, those who agreed to participate in the study were asked to fill out the forms in a quiet room within the hospital. A “do not to enter” sign was hung on the door of the room. Each woman was taken into the room with her baby. The researcher took care of the baby while the woman filled out the forms. It took the women an average of 15–20 min to fill out the forms.

## Data Collection Tools

The Personal Information Form, Postpartum Depression Screening Scale (PDSS), Spiritual Well-Being Scale (SWBS) and Spiritual Care Needs Inventory (SCNI) were used as data collection tools. The psychometric tools used in this study (PDSS, SWBS, and SCNI) were carefully selected for their conceptual clarity and validated use in the Turkish population. These scales contain mental health items that conceptually contaminate the construct of spirituality, as outlined by recent critiques (Bambling, 2024; Koenig & Carey, 2024, 2025). The full list of scale items in both Turkish and English versions is presented in Appendix 1, 2 and 3.

Therefore, the analysis focused on the relationships between the subscales of the SWBS. However, the associations found between postpartum depression and the harmony with nature and anomie subscales were not considered methodologically meaningful due to potential construct contamination. Only the relationship between the transcendence subscale, which specifically reflects spiritual aspects, and postpartum depression was considered significant.

### Personal Information Form

The form, prepared by the researchers based on a literature review, consists of 25 items designed to assess women's sociodemographic characteristics (e.g., age, height, weight, education level, monthly income), obstetric characteristics (e.g., number of pregnancies, type of birth, breastfeeding status), and spiritual practices (e.g., religious beliefs, religious practices and rituals). This form was developed in reference to previous studies (Wu et al., 2016; Zimmer et al., 2016).

### Postpartum Depression Screening Scale (PDSS)

The PDSS developed by Beck and Gabla in 2000 has 35 items each of which describes how the mother feels after birth (Beck & Gable, 2000). The validity and reliability of the Turkish version of the PDSS was conducted by Karaçam and Kitis (2008). Responses given to the items are rated on a five-point scale. The higher the score obtained from the PDSS is the higher the woman’s postpartum depression level is. The PPDS scale is designed to screen for depression risk and should not be used for diagnostic purposes.

Karaçam and Öner conducted the cut-off value studies of the Turkish version of the PDSS in 2008 (Karaçam & Öner, 2008). A total score of 55 or more indicates that women are more likely to have minor or major depression risk after birth. When the woman fills out the scale, she is asked to rate each item to indicate the situation that best describes the feelings she has experienced for the last two weeks, from 1 (strongly disagree) to 5 (strongly agree). The minimum and maximum possible scores that can be obtained from the total PDSS are 35 and 175, respectively.

The Turkish version of the PDSS includes the following six subscales: Emotional Lability (items 2, 4, 5, 9, 10, 12, 17, 19, 24, 31, 32), Contemplating Harming Oneself (items 7, 11, 14, 18, 21, 26, 27, 28, 35), Sleeping Disturbances (items 1, 15, 16, 22), Guilt (items 6, 13, 34), Eating Disturbances (items 3, 8, 29) and Anxiety (items 23, 25, 30). The Cronbach’s alpha internal consistency coefficient of the PDSS was 0.94 in its Turkish version (Karacam & Kitis, 2008), and 0.97 in this study.

### Spiritual Well-Being Scale (SWBS)

The SWBS was developed by Ekşi and Kardaş (2017) to determine how adults understand life and live it with its personal, social and transcendental aspects, based on their own values and interpretations. The scale consists of 29 items and the following three subscales: Transcendence (devotion to a divine power) (items 1, 4, 5, 8, 9, 12, 13, 16, 17, 20, 21, 24, 25, 27, 29), Harmony with Nature (items 2, 6, 10, 14, 18, 22, 28) and Anomie (meaning of life) (items 3, 7, 11, 15, 19, 23, 26). Each item is scored from 1 (not at all suitable for me) to 5 (very suitable for me). Of the 29 items in the SWBS, 22 are positively keyed items (items 1, 2, 4, 5, 6, 8, 9, 10, 12, 13, 14, 16, 17, 18, 20, 21, 22, 24, 25, 27, 28, 29), and seven are negatively keyed items (items 3, 7, 11, 15, 19, 23, 26).

The minimum and maximum scores that can be obtained from the SWBS are 29 and 145, respectively. As the score increases, so does the level of spiritual well-being. The Cronbach’s alpha value of the SWBS was 0.88 both in Ekşi and Kardaş's (2017) study and in the present study (Ekşi & Kardaş, 2017).

### Spiritual Care Needs Inventory (SCNI)

The SCNI was developed by Wu et al. (2016). The validity and reliability study of the Turkish version of the SCNI was conducted by Günay İsmailoğlu et al. (2019). The SCNI questions patients' spiritual care needs. The SCNI could be administered to all patients regardless of their religious beliefs and the reason for hospitalization.

The SCNI consists of 21 items each of which questions patients' potential spiritual care needs. Patients are asked to rate their spiritual care need in each item on a 5-point Likert type scale ranging from 1 to 5 (1 = Not at all necessary, 2 = Not necessary, 3 = Doesn't matter, 4 = Necessary, 5 = Absolutely necessary). The higher the mean score obtained from the total SCNI is the higher the level of the patient’s need for spiritual care.

The minimum and maximum possible scores that can be obtained from the scale are 21 and 105, respectively. The SCNI consists of the following two subscales: "Meaning and Hope" and "Caring and Respect". While the items in the Meaning and Hope Subscale (items 1, 2, 3, 4, 5, 6, 7, 8, 9, 10, 11, 12, 14) question one’s spiritual well-being towards self, nature and environmental factors, the items in the Caring and Respect Subscale (items 13, 15, 16, 17, 18, 19, 20, 21) question the person’s relationships with other people. The Cronbach's alpha value of the SCNI was 0.96 in Wu et al.'s study (Wu et al., 2016), 0.94 in Günay İsmailoğlu et al.’s study (Günay İsmailoğlu et al., 2019) and 0.96 in the present study.

## Ethics Declarations

Before the study was conducted, ethical approval was obtained from Selçuk University Faculty of Health Sciences Non-Interventional Clinical Research Ethics Committee (Decision date: April 27, 2022, Decision number: 2022/346). After receiving detailed information about the study, participants were invited to participate voluntarily and informed verbal consent was obtained. The study was carried out in accordance with the ethical standards established in the Declaration of Helsinki. During the collection of study data, of the participating women, those whose PPDS score was 55 and above (36.5%) were referred to specialists to receive psychological support.

### Data Analysis

The analysis of the data obtained from the study was analyzed on the computer using the Statistical Package for Social Science 29 (SPSS 29.0) program. Whether the data were normally distributed was checked with Skewness and Kurtosis values. Skewness and Kurtosis values ranging between − 2 and + 2 indicate that the data were normally distributed (Tabachnick & Fidell, 2013). Because the data in the study were normally distributed, parametric tests were used for analysis.

Descriptive variables were presented as number, percentages and arithmetic mean. In comparing descriptive variables with scale scores, independent samples t-test was used if there were two groups, and One-Way ANOVA (Analysis of Variance) was used if there were three or more groups.

The relationship between the PDSS, and SWBS and SCNI was checked using the Pearson Product Moment Correlation Technique. As the total score of the SWBS scale was found to overlap conceptually with the total PDSS score, it was excluded from the analysis to avoid contamination. Instead, the SWBS subscale scores were used in the correlation, regression and structural equation modelling (SEM) analyses.

The Forward model was used in linear regression analysis to examine the factors related to the PDSS. In the Forward model, variables with the highest correlation with the PDSS are included in the model (Xie et al., 2020). SEM was performed using IBM SPSS AMOS (version 26.0). Path analysis was conducted within the SEM framework to examine the mediating effects of the SWBS and SCNI subscales in the relationship between PDSS total score.

The significance level was accepted as *p* < 0.05. This study followed the STROBE recommendations for reporting. Statistical analyses were reviewed in collaboration with a professional statistician.

## Results

The mean age of the participants was 27.54 ± 5.67 (min = 18, max = 51) years. Their mean Body Mass Index (BMI) value was 27.17 ± 4.30. Of the participants, approximately 55% were secondary school graduates, 22.1% worked in an income-generating job, and 68.8% perceived their income level as medium. As for their obstetric characteristics, of them, 39.1% were primiparous, 22.3% had never given birth before, 49.1% gave birth vaginally, and 90.7% were within the first six months of postpartum. Of them, while 31.4% had problems with breastfeeding, 62.8% exclusively breastfed their babies.

The comparison of the scores the participants obtained from the total PDSS and their descriptive and obstetric characteristics demonstrated that of the participants, those whose perceived monthly income level was low obtained statistically higher scores than did those whose perceived monthly income level was medium or high (*p* < 0.001). The mean PDSS score of the primiparous participants was higher than was that of the multiparous participants and the difference between them was statistically significant (*p* = 0.006). The participants who experienced mental distress in the postpartum period obtained statistically higher scores than did those who did not experience mental distress (*p* = 0.008).

Those whose perceived monthly income level was high obtained statistically significantly higher scores from the total SWBS than did those whose perceived monthly income level was low (*p* = 0.011). Those who exclusively breastfed their babies obtained statistically significantly higher scores from the total SWBS than did those who fed their babies with breast milk and formula, only complementary food or only formula (*p* < 0.001). Those who experienced mental distress in the postpartum period obtained statistically significantly lower scores from the total SWBS than did those who did not experience mental distress (*p* = 0.037). Those who worked in an income-generating job (*p* = 0.049), those whose perceived monthly income level was high (*p* < 0.001), those who were smokers (*p* < 0.001) and those who had mental problems in the postpartum period (*p* < 0.001) obtained statistically higher scores from the Spiritual Care Needs Inventory than did other participants (Table 1).

**Table 1:** Comparison of the participants' scale scores in terms of their descriptive and obstetric characteristics (N = 430)

Variables	n	%	PDSS total score mean ± SD	SWBS total score mean ± SD	SCNI total score mean ± SD
*Age groups*					
18–30 age group	313	72.8	57.10 ± 26.82	124.79 ± 13.56	63.36 ± 21.86
31–51 age group	117	27.2	59.05 ± 26.70	122.79 ± 13.75	63.93 ± 20.27
**T**			− 0.669	1.356	− 0.243
***P***			0.504	0.176	0.808
*BMI groups*					
18.5–24.99 kg/m^2^ (Normal)	147	34.2	58.30 ± 25.26	123.19 ± 14.39	65.25 ± 20.71
25–29.99 kg/m^2^ (Over weight)	175	40.7	54.60 ± 23.92	125.78 ± 12.41	62.88 ± 21.19
30 and over kg/m^2^ (Obese)	108	25.1	61.63 ± 32.25	123.20 ± 14.32	62.18 ± 22.73
**F**			2.390	1.884	0.770
***p***			0.093	0.153	0.464
*Educational status*					
Elementary school (First 8 years)	38	8.8	60.52 ± 38.51	120.86 ± 13.78	68.55 ± 21.86
High school (Next 4 years)	236	54.9	57.60 ± 25.80	123.80 ± 13.40	61.98 ± 21.34
Higher education (Undergraduate, postgraduate, doctorate)	156	36.3	56.98 ± 24.84	125.75 ± 13.83	64.62 ± 21.30
**F**			0.267	2.262	1.870
***p***			0.766	0.105	0.155
*Working in an income-generating job*					
Yes	95	22.1	62.23 ± 32.51	123.35 ± 14.95	67.33 ± 21.47
No	335	77.9	56.33 ± 24.80	124.50 ± 13.24	62.43 ± 21.31
**t**			1.638	− 0.723	1.974
***p***			0.058	0.470	**0.049**
*Family type*					
Nuclear family	368	85.6	56.58 ± 25.45	124.61 ± 13.55	63.58 ± 21.35
Extended family	62	14.4	63.87 ± 33.10	122.06 ± 13.98	63.12 ± 21.96
**t**			− 1.989	1.367	0.156
***p***			0.051	0.086	0.438
*Perceived monthly income level*					
Income less than expenses^a^	65	15.1	69.69 ± 34.20	121.21 ± 12.29	63.93 ± 20.08
Income equal to expenses^b^	296	68.8	55.89 ± 25.06	124.01 ± 14.24	61.21 ± 21.24
Income more than expenses^c^	69	16.1	53.73 ± 22.96	128.13 ± 11.21	73.02 ± 21.02
**F**			8.212	4.523	8.841
***p***			** < 0.001**^**a>b,c**^	**0.011**^**c>a**^	** < 0.001**^**c>a,b**^
*Smoking*					
Yes	47	10.9	56.14 ± 23.42	124.59 ± 12.73	73.51 ± 20.70
No	383	89.1	57.81 ± 27.17	124.20 ± 13.75	62.29 ± 21.20
**t**			− 0.403	0.183	3.430
***p***			0.687	0.427	** < 0.001**
*Presence of a chronic disease*					
Yes	44	10.2	54.22 ± 24.31	126.31 ± 14.86	64.04 ± 22.68
No	386	89.8	58.02 ± 27.04	124.01 ± 13.48	63.46 ± 21.30
**t**			− 0.891	1.062	0.171
***p***			0.373	0.289	0.864
*Having problems during pregnancy*					
Yes	76	17.7	59.71 ± 26.29	124.48 ± 12.11	67.31 ± 21.48
No	354	82.3	57.19 ± 26.88	124.20 ± 13.95	62.70 ± 21.34
**t**			0.744	0.166	1.706
***p***			0.457	0.868	0.089
*The number of pregnancies*					
Primiparous	168	39.1	62.20 ± 29.16	125.04 ± 13.38	63.87 ± 21.19
Multiparous (two or more)	262	60.9	54.70 ± 24.73	123.74 ± 13.79	63.29 ± 21.59
**t**			2.758	0.963	0.274
***p***			**0.006**	0.168	0.784
*The number of previous births*					
None	96	22.3	62.22 ± 36.41	126.45 ± 14.42	62.50 ± 22.96
1 (One)	186	43.3	57.70 ± 23.67	124.04 ± 13.38	63.44 ± 20.66
≥ 2 (two or more)	148	34.4	54.56 ± 22.49	123.08 ± 13.33	64.28 ± 21.43
**F**			2.401	1.832	0.203
***p***			0.092	0.161	0.816
*Mode of the last birth*					
Vaginal birth (with or without intervention)	211	49.1	55.35 ± 23.82	124.97 ± 13.86	61.89 ± 22.24
Caesarean section	219	50.9	59.84 ± 29.22	123.55 ± 13.39	65.09 ± 20.51
**t**			− 1.742	1.076	− 1.551
***p***			0.082	0.141	0.061
*Postpartum period*					
The first six months	390	90.7	57.15 ± 26.54	124.52 ± 13.22	63.56 ± 21.63
Between the seventh and twelfth months	40	9.3	62.30 ± 28.83	121.62 ± 17.13	63.07 ± 19.42
**t**			− 1.157	1.038	0.138
***p***			0.248	0.305	0.890
*Having problems in breastfeeding*					
Yes	135	31.4	57.74 ± 24.34	125.93 ± 13.27	65.28 ± 21.61
No	295	68.6	57.58 ± 27.85	123.48 ± 13.74	62.71 ± 21.31
**t**			0.054	1.735	1.158
***p***			0.957	0.083	0.247
*Breastfeeding method*					
Exclusive breastfeeding^a^	270	62.8	56.58 ± 27.33	125.02 ± 12.63	62.41 ± 21.86
Breast milk and complementary food^b^	60	14.0	65.20 ± 28.99	118.16 ± 16.76	68.58 ± 19.89
Others (breast milk and formula, only complementary food or only formula))^c^	100	23.2	55.94 ± 23.12	125.82 ± 13.33	63.47 ± 20.85
**F**			2.827	7.276	2.046
***p***			0.060	** < 0.001**^**a,c>b**^	0.131
*Experiencing mental distress during the postpartum period*					
Yes	126	29.3	62.92 ± 26.65	122.11 ± 14.71	70.09 ± 21.13
No	304	70.7	55.44 ± 26.55	125.13 ± 13.08	60.79 ± 20.97
**t**			2.657	− 2.096	4.175
***p***			**0.008**	**0.037**	** < 0.001**

*PDSS* Postpartum Depression Screening Scale, *SWBS* Spiritual Well-Being Scale, *SCNI* Spiritual Care Needs Inventory, In the table, values with a *p*-value less than 0.05 are highlighted in bold

Of the participants, 98.6% were Muslims. The religious practices performed by the participants when they had problems in the postpartum period included saying prayers (32.3%), performing prayers (19.3%), reading the Quran (17.8%), performing ablution (washing hands, arms, face, and feet before praying) (16.7%), fulfilling the Sunnah (practices recommended by Prophet Muhammad), wearing an amulet, and counseling a religious leader (13.9%). Methods the participants used to cope with the problems experienced in the postpartum period were as follows: taking a walk in the nature (31.3%), listening to music (28.6%), watching television (25.4), and engaging in other practices such as sleeping, yoga, and meditation (14.7%).

Of the participants, those who resorted to religious practices in case they had a problem in the postpartum period obtained statistically lower scores from the PDSS than did those who did not seek refuge in religion (*p* = 0.035). Of the participants, those who performed religious practices in case they experienced a problem in the postpartum period (*p* < 0.001), those who were supported by health professionals when they performed religious practices (*p* < 0.001) and those who expected moral support from health professionals (*p* = 0.017) obtained statistically significantly higher scores from the total SWBS than did the other participants. Of the participants, those who prayed and asked God for help when they experienced a problem during the postpartum period (*p* = 0.023), those who were not supported by health professionals in their religious practices (*p* < 0.001), and those who did not expect moral support from health professionals (*p* = 0.006) obtained statistically higher scores from the Spiritual Care Needs Inventory than did the other participants (Table 2).

**Table 2:** Comparison of the relationship between the participants' postpartum spiritual practices and the scores they obtained from the Scales (N = 430)

Variables	n	%	PDSS total score Mean ± SD	SWBS total score Mean ± SD	SCNI total score Mean ± SD
*Taking refuge in religion in case a problem occurs*					
Yes	395	91.9	56.43 ± 25.07	125.18 ± 13.07	63.46 ± 21.08
No	35	8.1	71.22 ± 39.35	113.74 ± 15.46	64.11 ± 25.18
**t**			− 2.185	4.243	− 0.171
***p***			**0.035**	** < 0.001**	0.864
*Asking for help from god by praying in case a problem occurs*					
Yes	417	97.0	57.64 ± 26.76	124.35 ± 13.58	63.10 ± 21.11
No	13	3.0	57.38 ± 28.07	120.92 ± 15.21	76.76 ± 27.32
**t**			0.035	0.894	− 2.276
***p***			0.972	0.372	**0.023**
*Health professionals' encouraging them to pray*					
Yes	173	40.2	57.16 ± 24.917	124.67 ± 13.23	61.42 ± 20.76
No	257	59.8	57.95 ± 27.99	123.96 ± 13.91	64.93 ± 21.77
**t**			− 0.302	0.530	− 1.671
***p***			0.763	0.596	0.096
*Health professionals' providing support when religious practices (e.g., saying/performing prayers, reading the Quran) are performed*					
*d*					
Yes	158	36.7	56.81 ± 26.12	127.00 ± 11.98	70.61 ± 21.37
No	272	63.3	58.11 ± 27.17	122.65 ± 14.28	59.40 ± 20.37
**t**			− 0.448	3.229	5.403
***p***			0.626	** < 0.001**	** < 0.001**
*Expecting moral support from health professionals*					
Yes	287	66.7	57.20 ± 25.76	125.35 ± 13.40	65.52 ± 20.97
No	143	33.3	58.50 ± 28.76	122.02 ± 13.85	59.50 ± 21.80
**t**			− 0.473	2.400	2.767
***p***			0.636	**0.017**	**0.006**

*PDSS* Postpartum Depression Screening Scale, *SWBS* Spiritual Well-Being Scale, *SCNI* Spiritual Care Needs Inventory, in the table, values with a *p*-value less than 0.05 are highlighted in bold

The scores the participants obtained from the total PDSS, SWBS and SCNI were 57.63 ± 26.77, 124.25 ± 13.63 and 63.52 ± 21.41, respectively. According to the PDSS cut-off value, 36.5% of the participants were at risk for minor or major depression. There was a statistically significant negative relationship between the participants' scores on the Postpartum Depression Screening Scale (PDSS) and the transcendence subscale of the Spiritual Well-Being Scale (SWBS) (r =  − 0.191, *p* < 0.01).

Although negative correlations were also observed with the harmony with nature and anomie subscales (r =  − 0.200 and r =  − 0.463, respectively; *p* < 0.01), these associations were not considered methodologically meaningful due to potential overlap with mental health constructs. In other words, a higher level of transcendence, reflecting spiritual orientation, was associated with a lower risk of postpartum depression. There was no significant relationship between the scores the participants obtained from the total PDSS and the scores they obtained from the total SCNI and its subscales (*p* > 0.05) (Table 3).

**Table 3:** Relationship between the scores the participants obtained from the total PDSS, SWBS, SCNI and their subscales (N = 430)

				SWBS subscales				SCNI subscales	
	Scales	Mean ± SD	SWBS total	Transcendence	Harmony with nature	Anomie	SCNI total	Meaning and hope	Caring and respect
	**Mean ± SD**		124.25 ± 13.63	66.78 ± 8.66	30.97 ± 3.77	26.49 ± 5.64	63.52 ± 21.41	37.26 ± 14.18	26.25 ± 8.58
			**r value**	**r value**	**r value**	**r value**	**r value**	**r value**	**r value**
	PDSS total	57.63 ± 26.77	− 0.369*	− 0.191*	− 0.200*	− 0.463*	0.035	0.067	− 0.023
PDSS subscales	Emotional lability	18.30 ± 9.26	− 0.365*	− 0.186*	− 0.169*	− 0.483	0.050	0.080	− 0.008
	Contemplating harming Oneself	12.24 ± 6.82	− 0.375*	− 0.212*	− 0.236*	− 0.421	0.009	0.061	− 0.079
	Sleeping disturbances	7.74 ± 3.73	− 0.240*	− 0.085	− 0.169*	− 0.337*	0.005	0.012	− 0.008
	Guilt	5.03 ± 2.88	− 0.285*	− 0.140*	− 0.145*	− 0.375*	0.065	0.091	0.012
	Eating disturbances	5.45 ± 2.79	− 0.294*	− 0.147*	− 0.143*	− 0.389*	0.042	0.050	0.023
	Anxiety	5.96 ± 2.88	− 0.253*	− 0.144*	− 0.112*	− 0.316*	0.009	0.013	0.001

*PDSS* Postpartum Depression Screening Scale, *SWBS* Spiritual Well-Being Scale, *SCNI* Spiritual Care Needs Inventory, *SD* Standard Deviation

^*^p < 0.01

A multivariate regression model was created to investigate the factors associated with the scores the participants obtained from the total PDSS. The model accounts for approximately 26% of the variation in the PDSS total score of the independent variables (R^2^ = 0.258, *p* < 0.016). Of the characteristics of the participants, perceived monthly income (t =  − 2.412, *p* = 0.016) and the number of pregnancies (t =  − 3.257, *p* = 0.001) and the scores they obtained from the total SWBS (t =  − 3.203, *p* = 0.001) and its Anomie Subscale (t =  − 6.792, *p* < 0.001) were the predictors of the scores they obtained from the PDSS. All predictors were found to be negatively associated with postpartum depression scores, indicating that higher income levels, a greater number of pregnancies, higher levels of spiritual well-being, and higher levels of anomie (meaning of life) were associated with lower PDSS scores (Table 4).

**Table 4:** Analysis of the factors related to the participants' PDSS Scores with linear regression analysis (N = 430)

Variables	β	t	*p*	%95 CI		Tolerance	VIF
Perceived monthly income level	− 4.919	− 2.412	**0.016**	− 8.929	− 0.910	0.964	1.038
The number of pregnancies	− 3.033	− 3.257	**0.001**	− 4.864	− 1.203	0.978	1.022
SWBS total	− 0.320	− 3.203	**0.001**	− 0.517	− 0.124	0.673	1.485
Anomie	− 1.647	− 6.792	** < 0.001**	− 2.124	− 1.170	0.669	1.496

R = 0.508, R^2^ = 0.258, [F(df regression, df residual), F(1, 425) = 36.870], (Durbin–Watson = 1.894 (*p* < 0.016)

Dependent Variable: The participants' Postpartum Depression Screening Scale (PDSS) total score, Independent Variables: The participants' perceived monthly income level, the number of pregnancies, mental distress in the postpartum period, taking refuge in religion in case a problem occurs, SWBS: Spiritual Well-Being Scale and Spiritual Well-Being Scale Subscales (Transcendence, Harmony with Nature, Anomie), In the table, values with a *p*-value less than 0.05 are highlighted in bold

The SEM model showed an acceptable fit to the data based on several goodness of fit indices. The chi-squared statistic and degrees of freedom indicated an adequate fit (χ^2^/df = 18.356/5 = 3.671). The goodness-of-fit indices supported the adequacy of the model, with a goodness-of-fit index (GFI) of 0.986 and an adjusted goodness-of-fit index (AGFI) of 0.941.

**Fig. 1:**
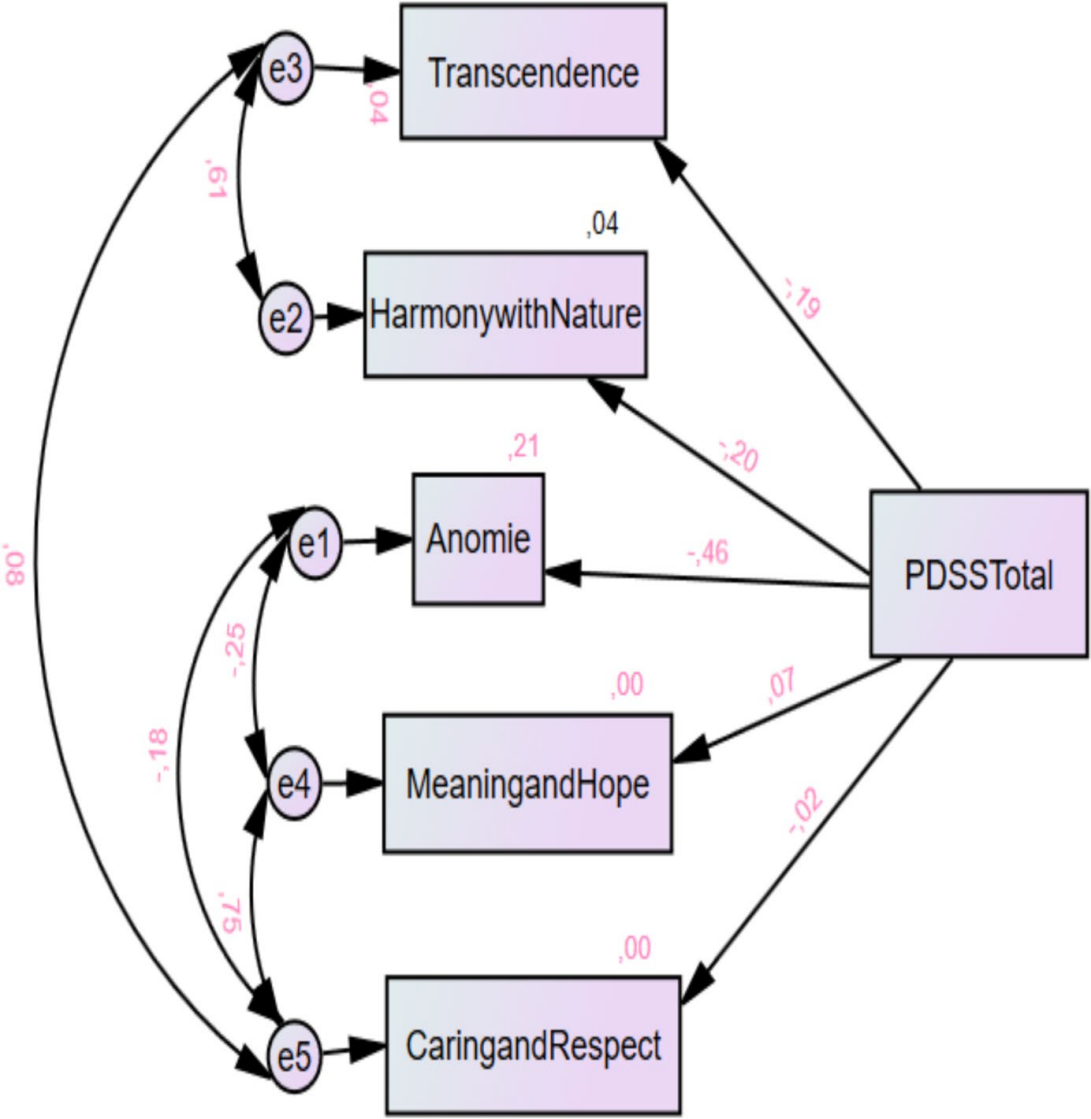
Path diagram of the SEM model showing the mediators of PDSS

The root mean squared error of approximation (RMSEA) was 0.079, suggesting a moderate fit. In addition, the comparative fit index (CFI) of 0.982 indicated a strong incremental fit. Regarding the parsimony-based fit, the parsimony normed fit index (PNFI) was 0.325. Finally, the expected cross-validation index (ECVI) was calculated as 0.117, providing insights into the model’s predictive validity (Fig. 1).

In the SEM, the transcendence (devotion to a divine power) subscale of the SWBS, exhibited a significant negative relationship with the PDSS (β =  − 0.192, *p* < 0.001). Although significant negative associations were also observed with the harmony with nature (β =  − 0.200, *p* < 0.001) and anomie (meaning of life) (β =  − 0.463, *p* < 0.001) subscales, these were not considered methodologically meaningful due to potential overlap with mental health constructs.

In contrast, the SCNI subscales “meaning and hope” (β = 0.067, *p* = 0.161) and “caring and respect” (β =  − 0.023, *p* = 0.631) did not serve as significant mediators of this relationship with the PDSS. These results show that only the transcendence (devotion to a divine power) subscale of spiritual well-being may play a distinct role in the postpartum depression process (Table 5).

**Table 5:** Examining the subscales of SWBS and SCNI as mediators in the relationship between the PDSS total score using SEM (N = 430)

Scales				β	Standardized β	Standard Error	R^2^	Critic Ratio	*p*
SWBS subscales	Harmony with Nature	←	PDSS Total	− 0.028	− 0.200	0.007	0.040	− 4.239	***
	Anomie	←	PDSS Total	− 0.098	− 0.463	0.009	0.215	− 10.824	***
	Transcendence	←	PDSS Total	− 0.062	− 0.192	0.015	0.037	− 4.059	***
SCNI subscales	Meaning and hope	←	PDSS Total	0.036	0.067	0.026	0.005	1.401	0.161
	Caring and respect	←	PDSS Total	− 0.007	− 0.023	0.015	0.001	− 0.481	0.631

*SWBS* Spiritual Well-Being Scale, SCNI Spiritual Care Needs Inventory

***p < 0.001

## Discussion

In the present study, 36.5% of the participants were at risk of minor or major depression in the postpartum period. In several studies, the prevalence of postpartum depression varies between 14 and 27% (Çankaya & Ataş, 2022; Liu et al., 2022; Özcan et al., 2017). In low-income countries, this prevalence varies between 20 and 40% (Coast et al., 2012; Parsons et al., 2012). Differences in PPD prevalence may have been due to the socioeconomic level of the societies where the studies were conducted, using different PPD screening scales, or not using of scales for diagnostic purposes.

In this study, of the participants, those whose perceived monthly income level was low were at risk of PPD. In a study conducted in Italy, the postpartum depression risk was approximately six times lower in women whose socioeconomic level was high (Cena et al., 2021). In a study conducted by Gebregziabher et al. (2020), the women who perceived their economic status as low were at a higher risk of postpartum depression (Gebregziabher et al., 2020).

Postpartum depression levels of the primiparous women were higher than were those of the multiparous women in study. Contrary to our study, in some studies, no relationship was determined between the number of pregnancies and postpartum depression (Gebregziabher et al., 2020; Zejnullahu et al., 2021). However, in the present study, the primiparous women had difficulty adapting to the postpartum period because they were less experienced, and therefore their risk of postpartum depression was high.

In the present study, of the participants, those who experienced mental distress in the postpartum period had higher postpartum depression levels. Not all women at risk of PPD may develop clinical symptoms (Cardoso & Fonseca, 2023), which indicates the importance of screening women for the risk of depression in the postpartum period. Spiritual beliefs strongly affect women in the postpartum period and can positively affect them spiritually (Cheadle & Dunkel Schetter, 2018).

In this study, 98.6% of the participants believed in Islam. Of them, those who had problems in the postpartum period mostly performed religious practices such as saying and performing prayers, and reading the Quran. In these participants, the risk of postpartum depression was lower when they had a problem in the postpartum period. It is known that listening to music during the birth process has a positive effect on the woman's postpartum mental health (Perkovic et al., 2021).

In another study, pregnant women’s listening to the Inshirah surah (a section of the Quran) in the first stage of labor significantly reduced their anxiety level (Kocak et al., 2022). A systematic review has found that religiosity is associated with a reduction in depression over time (Braam & Koenig, 2019). In their study (2016), Clements et al. reported that women with low religious commitment were at risk of depression (Clements et al., 2016). In Keefe et al.’s qualitative study (2016), the women in the postpartum period stated that they found peace in church, that they got rid of stress by praying, and that they asked for God's help (Keefe et al., 2016).

However, as women spend more time caring for the baby, their participation in religious practices may decrease. A reduced focus on spirituality has been suggested as a potential factor in postpartum mood disorders and in a diminished sense of self (Crowther et al., 2021). Several studies have suggested a possible relationship between religiosity, spirituality, and postpartum depression (Jacobs et al., 2012; Sternthal et al., 2010).

There are theoretical differences between religiosity and spirituality; thus, these concepts should be evaluated separately. In the present study, a negative relationship was found between participants’ total PDSS scores and their scores on the SWBS subscales. Notably, among these subscales, only transcendence-which reflects a distinctively spiritual dimension related to one’s devotion to a higher power-can be considered a meaningful correlate of postpartum depression, due to its relative independence from general mental health constructs.

The results suggest that lower levels of transcendence are significantly associated with higher levels of postpartum depression, highlighting the potential influence of spiritual orientation on maternal mental health. Similarly, both religiosity and spirituality predict lower depressive symptoms in the first year postpartum, with psychosocial resources mediating these relationships (Cheadle & Dunkel Schetter, 2018). In Akbari et al.’s study (2020), depressed mothers obtained lower scores from the SWBS (Akbari et al., 2020). These results indicate that spirituality may be an important support resource for preventing depression in the postpartum period.

In the present study determined, there was no relationship between the women's spiritual care needs and their postpartum depression levels. Spiritual care needs have generally been evaluated in the care of inpatients (Günay İsmailoğlu et al., 2019). In addition, spiritual care needs are complex and multidimensional (Seddigh et al., 2016). The study was conducted with women in the first 12 months postpartum. Spiritual care needs of women in the early postpartum period may be different.

## Limitations

The strength of the study is that its sample included a good number of women in the postpartum period. One of the limitations of the study is that the women's depression risk was determined based on self-report. The results provide screening information and clinical interviews are required for the diagnosis of depression. Another limitation of the study is that the data were obtained from the women in a province in the Anatolia Region of Turkey.

Therefore, the results are applicable only to the women surveyed and they cannot be generalized to all pregnant women. An additional limitation of the study is that it was not possible to determine whether the participants' engagement in spiritual practices was situational (in response to distress or problems) or a part of their routine religious behavior. Future studies may consider distinguishing between reactive and habitual spiritual practices.

## Conclusion and Recommendations

Of the participants, 36.5% were at risk of postpartum depression. Those whose perceived monthly income level was low, those who were primiparous and those who experienced mental distress in the postpartum period had higher depression levels. Another important result of the study is that the risk of postpartum depression decreased as the participants' level of transcendence, which reflects their devotion to a higher power, increased.

It is known that women are at risk of depression in the postpartum period. However, women's spirituality levels which affect their depression symptoms are not investigated. To help prevent postpartum depression, health professionals should assess women's spiritual well-being as part of maternal care during the antepartum and postpartum periods.

Additionally, women should be encouraged to engage in spiritual practices aligned with their beliefs, such as relaxation techniques, meditation, or prayer, to promote emotional well-being throughout pregnancy, childbirth, and the postpartum period. Incorporating individual spiritual and cultural needs into maternal care planning can further support women's psychological resilience and overall health.

## Appendix 1

See Table

**Table 6:** Turkish and English Versions of the postpartum depression screening scale items

Doğum Sonu Depresyon Tarama Ölçeği	Postpartum Depression Screening Scale
Türkçe Maddeler	English Items
1. Bebeğim uyurken bile uyumakta zorlanıyorum	1. Had trouble sleeping even when my baby was asleep
2. Kendimi tamamen yalnız hissediyorum	2. Felt all alone
3. Hiç sebep yokken çok fazla ağlıyorum	3. Cried a lot for no real reason
4. Herhangi bir şeye yoğunlaşamıyorum	4. Could not concentrate on anything
5. Artık kendi kendimi tanıyamıyorum	5. Did not know who I was anymore
6. Bir anne olarak kendimi yetersiz hissediyorum	6. Felt like a failure as a mother
7. Ölmenin daha iyi olacağını düşünmeye başladım	7. Started thinking that I would be better off dead
8. İştahımı kaybettim	8. Lost my appetite
9. Kendimi ağır bir yükün altında ezilmiş gibi hissediyorum	9. Felt really overwhelmed
10. Asla tekrar mutlu olamayacağımdan korkuyorum	10. Was scared that I would never be happy again
11. Aklımı kaybediyormuşum gibi hissediyorum	11. Felt like I was losing my mind
12. Kendi kendime yabancılaştığımı hissediyorum	12. Felt as though I had become a stranger to myself
13. Birçok annenin benden daha iyi olduğunu hissediyorum	13. Felt like so many mothers were better than me
14. Ölümün bu yaşanan kâbustan kurtulmanın tek yolu olduğunu düşünüyorum	14. Have thought that death seemed like the only way out of this living nightmare
15. Gece yarısı kendiliğinden uyanıyorum ve tekrar uyumakta güçlük çekiyorum	15. Woke up on my own in the middle of the night and had trouble getting back to sleep
16. Yerimden sıçradığımı hissediyorum	16. Felt like I was jumping out of my skin
17. Duygularımın alt üst olduğunu hissediyorum	17. Felt like my emotions were on a roller coaster
18. Çıldırdığımı düşünüyorum	18. Thought I am going crazy
19. Asla tekrar eskisi gibi normal olamayacağımdan korkuyorum	19. Was afraid that I would never be my normal self again
20. Bebeğime gerektiği kadar çok sevgi hissedemediğim için, suçluluk duyuyorum	20. Felt guilty because I could not feel as much love for my baby as I should
21. Kendime zarar vermek istiyorum	21. Wanted to hurt myself
22. Geceleyin uykuya dalmak için uzun süre dönüp duruyorum	22. Tossed and turned for a long time at night trying to fall asleep
23. Bebeğim ile ilgili en küçük şeyde bile aşırı derecede endişeleniyorum	23. Got anxious over even the littlest things that concerned my baby
24. Çok fazla huzursuzum	24. Have been very irritable
25. Basit bir kararı vermede bile zorlanıyorum	25. Had a difficult time making even a simple decision
26. Normal olmadığımı hissediyorum	26. Felt like I was not normal
27. Bebeğime karşı düşündüklerimi ve hissettiklerimi saklamak zorundaymışım gibi hissediyorum	27. Felt like I had to hide what I was thinking or feeling towards the baby
28. Bebeğimin bensiz daha iyi olacağını hissediyorum	28. Felt that my baby would be better off without me
29. Yemem gerektiğini biliyorum, fakat yiyemiyorum	29. Knew I should eat but I could not
30. Sürekli yürümek ya da hareket etmek zorundaymışım gibi hissediyorum	30. Felt like I had to keep moving or pacing
31. Öfkeden patlamaya hazır olduğumu hissediyorum	31. Felt full of anger ready to explode
32. Bir işe yoğunlaşmakta zorlanıyorum	32. Had difficulty focusing on a task
33. Kendimi gerçekmiş gibi hissedemiyorum	33. Did not feel real
34. İstediğim gibi bir anne olmadığımı hissediyorum	34. Felt like I was not the mother I wanted to be
35. Sadece bu dünyadan ayrılmak istiyorum	35. Just wanted to leave this world

6.

## Appendix 2

See Table

**Table 7:** Turkish and English versions of the Spiritual Well-Being Scale items

Spiritüel İyi Oluş Ölçeği	Spiritual Well-Being Scale
Türkçe Maddeler	English Items
1. İlahi bir güce bağlı olmak bana güven verir	1. Being connected to a divine power gives me confidence
2. Doğaya saygı duyulması gerektiğini düşünürüm	2. I believe that nature should be respected
3. Hayata dair bir hoşnutsuzluk duygusu hissederim	3. I feel a sense of dissatisfaction with life
4. Bir problemle karşılaştığımda Allah’ın yardımını hissederim	4. I feel God's help when I encounter a problem
5. Allah’ın gizli ve açık tüm duygu ve düşüncelerimi bildiğine inanırım	5. I believe that God knows all my hidden and open feelings and thoughts
6. Bütün canlıların saygıyı hak ettiğini düşünürüm	6. I believe that all living beings deserve respect
7. Hayatımda büyük bir boşluk var	7. There is a great void in my life
8. Günlük hayatta Allah’ın kudretine şahit olurum	8. I witness God's power in daily life
9. Allah’ın beni sevdiğine ve önemsediğine inanırım	9. I believe that God loves and cares about me
10. Yeryüzündeki tüm canlılara iyi davranırım	10. I treat all living beings on earth well
11. Hayattan zevk almam	11. I don't enjoy life
12. Hayatımın her anında Allah’ın varlığını hissederim	12. I feel God's presence in every moment of my life
13. Daha güçlü bir varlığa sığınma duygusu beni rahatlatır	13. The feeling of taking refuge in a higher being comforts me
14. Kendimi doğanın bir parçası olarak görürüm	14. I see myself as a part of nature
15. Hayatımın amacını halen bulabilmiş değilim	15. I haven't been able to find the purpose of my life yet
16. Yaşadığım her olayda bir hayır olduğuna inanırım	16. I believe there is a reason for everything I experience
17. İnancım, nasıl bir hayat süreceğime dair bana yol gösterir	17. "My faith guides me on how to live my life."
18. Yeryüzündeki bütün canlıların hakları benim için önemlidir	18. The rights of all living beings on earth are important to me
19. Sorunlarımı çözmeye nereden başlayacağımı bilemem	19. I don't know where to start solving my problems
20. Yalnız kaldığımda Allah’ı ve yarattıklarını düşünürüm (tefekkür ederim)	20. When I am alone, I think about God and His creations (I contemplate)
21. İnanç ve değerlerim, zorluklar karşısında dayanabilme gücümü arttırır	21. "My faith and values increase my ability to endure difficulties."
22. Doğayla uyum içinde yaşarım	22. I live in harmony with nature
23. Zorluklar yaşadığımda bunalmış hissederim	23. I feel overwhelmed when I experience difficulties
24. İnancım, yaşadığım sıkıntılarda dahi olumlu tarafların olabileceğini görmemi sağlar	24. My faith allows me to see that there can be positive aspects even in the troubles I experience
25. Hayatta hiçbir şey sebepsiz değildir	25. Nothing in life is without a reason
26. Hayatın beni mutsuz eden olaylardan ibaret olduğunu düşünürüm	26. I think that life is just a series of events that make me unhappy
27. Her şeyin elimde olmadığını bilmek üzüldüğüm olaylar karşısında bir teselli kaynağıdır	27. Knowing that everything is not in my control is a source of comfort in the face of events that make me sad
28. Yeryüzündeki her doğal varlığın eşsiz olduğuna inanırım	28. I believe that every natural entity on earth is unique
29. Dünya hayatının geçici olduğuna inanmak beni hırslarımdan arındırır	29. Believing that worldly life is temporary frees me from my ambitions

7.

## Appendix 3

See Table

**Table 8:** Turkish and English Versions of the Postpartum Depression Screening Scale items

Spiritüel Bakım Gereksinimleri Ölçeği Türkçe Maddeler	Spiritual Care Needs Inventory (SCNI) English Items
1. Dünya ile barış içinde olmak için rehberliğe	1. Guidance to being at peace with the world
2. Kendimi ifade edebilmemde sanat ve yaratıcılığı kullanmak için rehberliğe	2. Guidance to use art and creativity for self-expression
3. Doğa ile ilgilenmem için rehberliğe	3. Guidance to connect with nature
4. Suçluluk duygusunun ortadan kaldırılmasına	4. Eliminating a sense of guilt
5. Hayatın anlam ve amacını bulmak için rehberliğe	5. Guidance to discover the meaning and purpose in life
6. Hayatta sıkıntılarla karşılaştığımda hayatın anlamını bulmak için rehberliğe	6. Guidance to find the meaning when facing troubles in life
7. İçinde bulunulan anı yaşamak için rehberliğe	7. Guidance to live in the moment
8. Güven duymak için rehberliğe	8. Guidance to find confidence
9. Manevi iletişime imkan sağlanması için rehberliğe	9. Allowing spiritual communication
10. İç huzuru bulmak için rehberliğe	10. Guidance to find inner peace
11. Hayatta umutlu olmak için rehberliğe	11. Guidance to gain a sense of hope in life
12. Cesaretlendirilmeye	12. Bringing me courage
13. Bir ibadethane bulmak için rehberliğe	13. Guidance to find a place of worship or a church
14. Bana iyilik duygusu kazandırılmasına	14. Bringing me a sense of well-being
15. Bana destek ve güven verilmesine	15. Supporting and reassuring me
16. Benimle etkileşim kurulmasına (karşılıklı konuşma, sohbet etme gibi)	16. Interacting with me (e.g., chat, talking)
17. Bana ilgi gösterilmesine	17. Showing concern for me
18. Mahremiyetime ve onuruma saygı gösterilmesine	18. Respecting my privacy and dignity
19. Bana dostluk ve kişilerarası ilişki kazandırılmasına	19. Bringing me friendship and interpersonal relationships
20. Korkularımı, endişelerimi ve sorunlarımı tartışmak ve keşfetmek için zaman tanınması ve dinlenilmesine	20. Listening to and allowing time to discuss and explore my fears, anxieties and troubles
21. Dini ve kültürel inançlarıma saygı duyulmasına	21. Respecting my religious and cultural beliefs

8.
